# In Situ Characterisation of Hydrogels via Dynamic Interface Printing

**DOI:** 10.1002/advs.202515106

**Published:** 2026-03-07

**Authors:** Callum Vidler, Michael Halwes, David J. Collins

**Affiliations:** ^1^ Department of Biomedical Engineering The University of Melbourne Melbourne Victoria Australia; ^2^ The Graeme Clark Institute The University of Melbourne Parkville Victoria Australia

**Keywords:** bioprinting, dynamic interface printing, hydrogel, light‐sheet, mechanical characterization

## Abstract

Hydrogels have become pivotal materials for tissue engineering, robotics, biomedical devices, and sensing applications due to their diverse material compositions and tunable mechanical properties. While significant effort has focused on developing novel manufacturing approaches such as extrusion bioprinting and light‐based fabrication methods, there has been limited work in real‐time characterisation of manufactured parts, which often requires tedious parameter optimization to achieve the desired structural resolution and material properties. Here, we demonstrate a high‐throughput approach based on Dynamic Interface Printing (DIP) that enables simultaneous in situ fabrication, mechanical characterisation, and volumetric quantification of centimeter‐scale hydrogel scaffolds within seconds. We establish automated stiffness‐seeking capabilities through a characterisation‐in‐the‐loop framework employing zero‐order optimization algorithms, achieving target elastic moduli within 3%–5% accuracy across diverse material formulations without prior knowledge of material properties or structural information. We further introduce a three‐dimensional nodal framework for volumetric grayscale lithography that generates spatially heterogeneous mechanical properties, demonstrating engineered nonlinear stress‐strain relationships through crosslinking density modulation. In addition, we implement orthogonal light‐sheet illumination coupled with machine learning segmentation algorithms, enabling real‐time layer‐wise structural reconstruction with >85% accuracy. This integrated methodology eliminates manual handling, automating part design and significantly shortening optimization timelines by providing real‐time quantitative feedback on morphology and mechanical properties.

## Graphical abstract

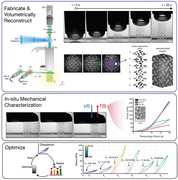

## 1 Introduction

Hydrogels constitute a distinctive class of materials owing to their close resemblance to native human tissues, and have therefore been widely employed in soft robotics [1, 2], microfluidics [3, 4], biosensing [5, 6,], and drug delivery [7, 8, 9]. Their broad tunability spanning mechanical properties [10, 11, 12, 13, 14], chemical composition [15, 16, 17, 18], photocrosslinking chemistry [19, 20, 21, 22], adhesion/attachment motifs [23, 24, 25], and biocompatibility [26, 27, 28, 29, 30], makes them particularly well suited to applications that demand compliant, tissue‐like environments. Hydrogels have therefore become foundational materials for tissue engineering, providing not only a cytocompatible microenvironment for cell growth and function, but also supporting the integration of multiscale structural complexity, including hierarchical organisation [16, 17, 31], vascular‐like networks [32, 33, 34, 35, 36, 37] and spatial stiffness gradients [38, 39, 40], thereby improving fidelity to native tissue architecture. More recently, bioprinting has emerged as a powerful route to both implement and expand this complexity by enabling readily programmable control over micro‐ and macroscale architecture through geometric design [32, 33, 35, 41], greyscale modulation [38, 40] and, and multi‐material compositions [18, 31, 42, 43, 44, 45].

Whilst the bulk of bioprinting has to date focused on extrusion‐based systems [46, 47, 48], the use of light‐based approaches has increasingly become more prevalent, providing the capacity for higher resolution and more physiologically relevant length scales to be readily created by patterning alone. For instance, techniques based on two‐photon polymerization (2PP) can readily create constructs with nanoscale features [49, 50, 51], with lower‐cost approaches such as projection micro‐stereolithography (PµSL) enabling single‐digit micron resolution [52, 53], with conventional DLP/LCD approaches still achieving physiologically relevant length scales [14, 33, 54]. Recently, much focus has been placed on creating systems capable of increasing structural throughput and control, driven by the desire to expand the translatability of these techniques. More recently, emphasis has been placed on improving throughput and process control to enhance the translational utility of light‐based fabrication. In this context, tomographic volumetric printing [17, 27, 55, 56, 57] and light‐sheet printing [44, 58, 59] have delivered substantial gains in fabrication rate due to simultaneous or serial fabrication of the entire construct, without relative fluid movement. In particular, tomographic volumetric printing has been used extensively in the context of biofabrication for investigating the importance of geometric design on tissue and cellular behaviour [41, 57], formulation‐driven cellular organisation [60], and material‐driven multi‐scale hierarchical control [36].

Other recent approaches such as Dynamic Interface Printing [16, 61] based on an acoustically modulated air‐liquid interface, as well as acoustically augmented variants of volumetric printing [62] or DLP [63] printing have shown that greater multiscale control can be achieved by combining light with acoustic fields to organise nonphotoresponsive constituents within photocrosslinkable materials or in some cases directly writing material with acoustics alone [64]. Despite a growing array of fabrication techniques, however, efforts are still required to streamline the characterisation and evaluation of hydrogel scaffolds, where ensuring the consistent and accurate replication of structural morphology and mechanical properties is critical for emerging high‐throughput screening applications based on printed hydrogel tissue models.

In this work, we present a high‐throughput biofabrication platform that integrates DIP‐based fabrication with in situ mechanical characterisation and volumetric quantification of centimetre‐scale constructs, achieved in as little as 60 s, including print time. While our previous work on Dynamic Interface Printing [61] established the technique as a rapid, high‐throughput 3D fabrication method, the present study extends these principles to realise a closed‐loop system for material and structural characterisation, enabling us to design the mechanical performance of printed hydrogels.

A primary limitation in soft hydrogel fabrication to date is the difficulty in assessing mechanical properties, as printed structures often deform under their own weight. This typically results in measurements that reflect bulk material behaviour rather than the mechanical response of the printed architecture. In this work, we show how our approach not only enables the generation of free‐standing hydrogel constructs, but permits in situ mechanical characterisation without premature gravitationally‐induced collapse of soft hydrogel materials. By embedding mechanical and volumetric characterisation directly within the fabrication process, we demonstrate automated, in situ mechanical evaluation with further capacity for stiffness‐seeking control, volumetric greyscale patterning, and on‐demand three‐dimensional reconstruction of hydrogel scaffolds, collectively establishing a pathway toward accurate, repeatable, and scalable fabrication of mechanically tailored biomaterials.

## 2 Results

### 2.1 Dynamic Interface Printing Approach

Dynamic Interface Printing (DIP) demonstrates distinct advantages over other established high‐throughput printing methodologies, including continuous liquid interface production (CLIP) [65, 66, 67], volumetric additive manufacturing (VAM) [41, 55, 56], and Xolography [44, 58, 59]. Notably, DIP permits the rapid fabrication of structures directly within arbitrary volumes accessible to the print head, such as multi‐well plates, whilst circumventing the reconstruction and material nuances traditionally imposed by volumetric systems. DIP employs a hollow print head incorporating a transparent optical window for axial projection and an integrated pressurization system. Upon immersion in photopolymer, cavity pressurization generates a controlled meniscus at the print head's extent. This air‐liquid interface serves as the fabrication boundary, where the absence of rigid structural contact eliminates mechanical adhesion, facilitating both high‐throughput fabrication and low structural stress, crucial for highly soft and fragile constructs.

Conventional light‐based fabrication methodologies typically require post‐fabrication removal of printed structures from their build platform or containment volume prior to mechanical characterisation. This presents challenges for hydrogel‐based materials with low elastic moduli (0.5–50 kPa), which are susceptible to deformation and damage during handling. Additionally, high‐aspect ratio or complex three‐dimensional architectures may deform or collapse during printing under their own weight. This mechanical instability complicates the deconvolution of structure‐specific mechanical properties from intrinsic material properties, as architectural features are compromised before characterisation can occur. As such, this work addresses these limitations by maintaining structural integrity during both fabrication and characterisation by preserving buoyant support.

**FIGURE 1:**
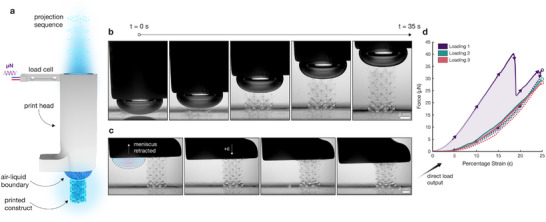
Overview of the dynamic interface printing mechanical characterisation system. (a), Illustration of the dynamic interface printing (DIP) approach, whereby an air–liquid boundary is formed at the extent of a submerged print head and used to rapidly create structures in situ. (b), Time series images of the DIP system printing a gyroid TPMS structure, showing the creation of a 10 mm tall construct in approximately 45 s. (c), Time series images during initial uniaxial compression of the TPMS lattice using the flat portion of the print head. (d), Realtime load cell output during uniaxial compression, showing the initial loading and collapse of the structure, followed by two secondary loading cycles.

To enable direct mechanical characterisation, we integrate a load cell with a DIP print head (Figure 1a), where this modification permits immediate force‐displacement measurements during printing. The process initiates with print head submersion and pressurization, establishing an air‐liquid meniscus at the print head's orifice. This meniscus is brought in contact with the base of the container, which establishes the first cured region of the target object with the application of light. To account for the slicing mismatch between a standard Cartesian voxel array and the curved surface formed by the air‐liquid meniscus, we utilise a convex slicing algorithm that approximates the interface profile by solving the Young‐Laplace equation using cubic Bézier curves [61, 68]. Following interface profile determination, nearest‐neighbour interpolation identifies voxels coincident with the meniscus throughout the print sequence. This computational approach addresses both steady‐state meniscus configurations and transient profiles during initial compression. A hydrogel construct is fabricated via continuous *z*‐axis translation The achievable print rate is primarily limited by the replenishment of uncured material beneath the meniscus and therefore depends strongly on the viscosity of the prepolymer solution. To mitigate this limitation, tuneable acoustic modulation of the confined air cavity can be used to generate propagating capillary waves at the air–liquid interface, which enhances interfacial streaming and significantly improves mass transport in more viscous formulations, as demonstrated previously [61]. For materials that undergo thermal gelation (e.g., gelatin‐based formulations), a temperature‐controlled, HEPA‐filtered enclosure maintains the resin at 37°C to ensure the material remains in a liquid state during fabrication.

Following fabrication, the printed scaffold is mechanically characterised in situ using a load cell integrated into the print head (Figure 1d). Uniaxial compression is applied by translating the print head toward the base of the container, while force and displacement are recorded concurrently from the load cell and the linear stage. Uniaxial compression is delivered via a rigid platform incorporated into the print head adjacent to the aperture (Figure 1c). A representative printing and mechanical testing process is captured in Supplementary Movie S1.

### 2.2 Mechanical Characterisation

**FIGURE 2:**
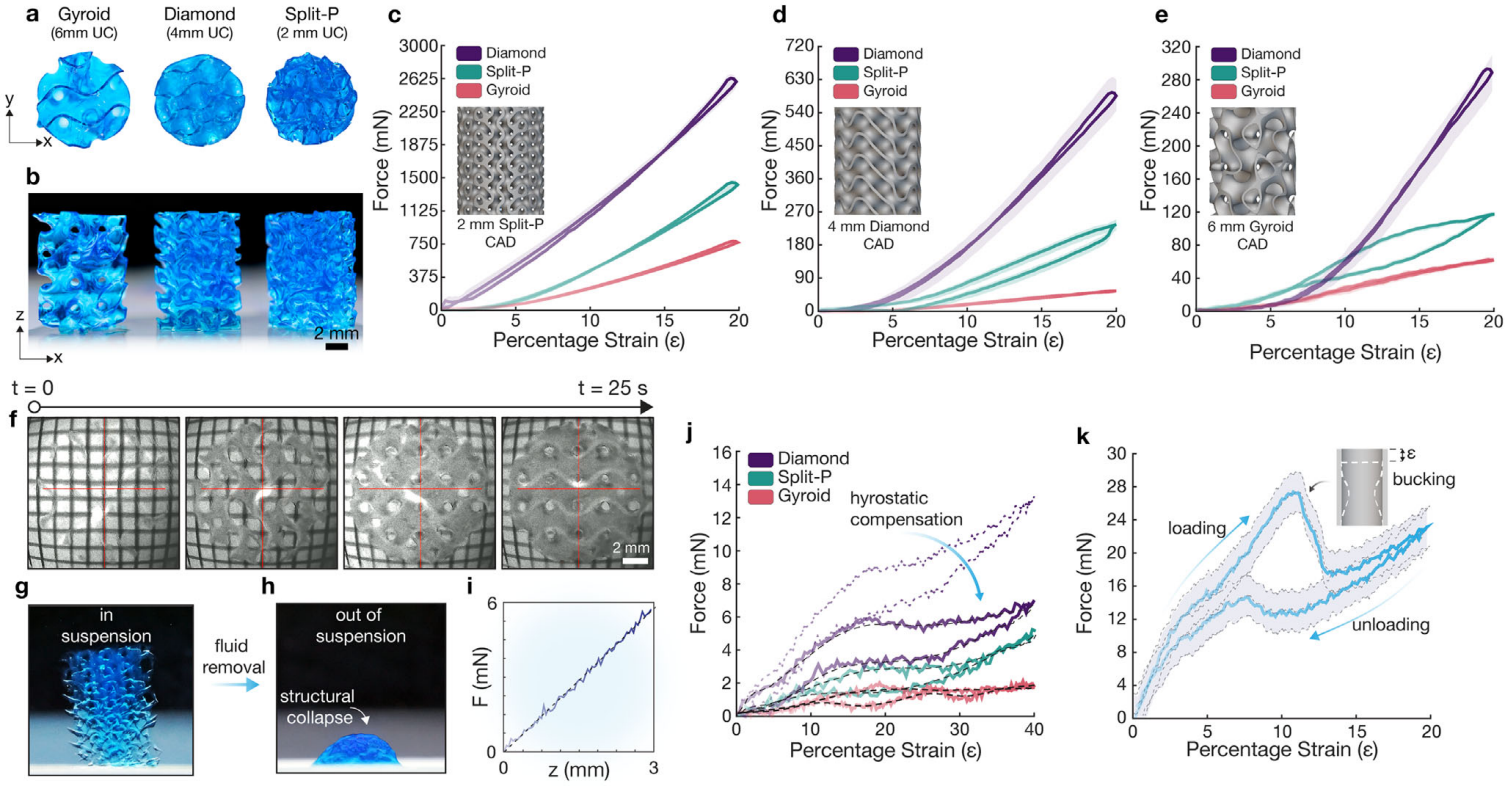
In situ mechanical characterisation of TPMS hydrogel structures. (a), Top‐down view of the three TPMS unit‐cell morphologies at 6‐, 4‐ and 2‐mm respectively comprised of 30% PEGDA. (b), Side view of the three 6 mm unit cell TPMS variants. (c–e), In situ mechanical characterisation of each 30% PEGDA TPMS variant, for increasing unit cell sizes of 2‐, 4‐ and 6‐mm (*n* = 3). (f), Real‐time coaxial imaging during fabrication of the 4 mm unit cell 10% PEGDA Gyroid. Image sequence lapses approximately 25 s. (g, h), 4 mm unit cell 10% PEGDA Gyroid in suspension and out of suspension respectively. (i), Linear hydrostatic calibration of material density. (j), Mechanical characterisation of the 4 mm TPMS variants utilising the 10% PEGDA formulation. Dashed and solid lines for the diamond lattice indicate the raw measured load and hydrostatic corrected load respectively. (k), Uniaxial compression and induced buckling of a thin‐walled cylindrical structure comprised of 10% PEGDA.

Here, we demonstrate the ability to examine complex, three‐dimensional structures exhibiting geometry‐dependent mechanical properties, which are governed by unit cell size and morphology. Three triply periodic minimal surface (TPMS) architectures—Gyroid, Diamond, and Split‐P—were fabricated with 2‐, 4‐, and 6‐mm unit cell dimensions from 30% w/v poly(ethylene glycol) diacrylate (PEGDA) (Figure 2a,b), with triplicate recordings (*n* = 3) captured for each of the 9 geometry configurations (Figure 2c–e). As expected, structural stiffness increased with decreasing unit cell size. When aggregated across all unit cell dimensions, we observed consistent differences between TPMS types. When normalized against the Diamond geometry, the Split‐P and Gyroid architectures exhibited approximately 2.5X and 6X lower stiffness, consistent with prior reports comparing geometry‐dependent variation in TPMS mechanical responses [69, 70]. Real‐time, top‐down fabrication monitoring employs coaxial imaging to verify architectural fidelity throughout the printing process (Figure 2f).

Low polymer‐content formulations typically yield highly compliant constructs, which makes conventional post‐fabrication mechanical testing prone to gravity‐induced deformation. Although such low‐stiffness structures can be fabricated with high fidelity while supported within the surrounding liquid bath (Figure 2g), removal for ex situ testing often results in collapse. This compromises the ability to separate intrinsic material properties from architecture‐dependent mechanical behaviour (Figure 2h), as measured responses largely reflect a collapsed gel mass rather than the designed topology. In contrast, our approach mitigates these limitations by performing fabrication and mechanical characterisation in situ, where the surrounding unpolymerized material provides buoyant support throughout.

A consequence of in‐fluid testing is that hydrostatic forces contribute to the measured load and must be removed to isolate the structure's true mechanical response (see Methods). This correction is particularly important for low‐modulus materials, where hydrostatic contributions can be comparable to, or exceed, the structural force. Accordingly, prior to printing, the print head is translated through a known vertical interval to acquire force–position pairs ({*z_i_*, *F_i_*}) (Figure 2i). Because the hydrostatic contribution varies linearly with depth, linear regression is used to estimate the hydrostatic gradient and thereby infer the formulation density prior to fabrication. During uniaxial compression, the predicted hydrostatic component is subtracted from the recorded load to deconvolve the structural force. After correction, the measured response reflects the intrinsic mechanical behaviour of the printed architecture independent of buoyancy effects (Figure 2j), enabling accurate measurement of low‐stiffness structural behaviour such as buckling and snap‐through, with sub‐millinewton sensitivity even in the presence of a supporting fluid (Figure 2k).

### 2.3 Stiffness Seeking & Greyscale Lithography

The capacity to spatially tailor the mechanical properties of photocurable materials has a multitude of applications spanning meta‐materials [38, 40], microfluidics [3, 4], 4D‐materials [71, 72] and biofabrication [39, 73]. In the case of biofabrication, greyscale approaches can be used to tailor the underlying ECM properties to control cellular organisation within 3D tissue scaffolds [74, 75]. The variability in the material moduli here is driven by the varying optical dosage, which determines the cross‐linking density. While previous approaches have focused on computational approaches to predict the resultant structures' mechanical properties [76, 77], accurate modelling of the complexity of these reactions, including the non linear material response, convolved optical dosage from multi‐layer objects [38], optical scattering and attenuation make purely numerical approaches highly challenging to optimise.

**FIGURE 3:**
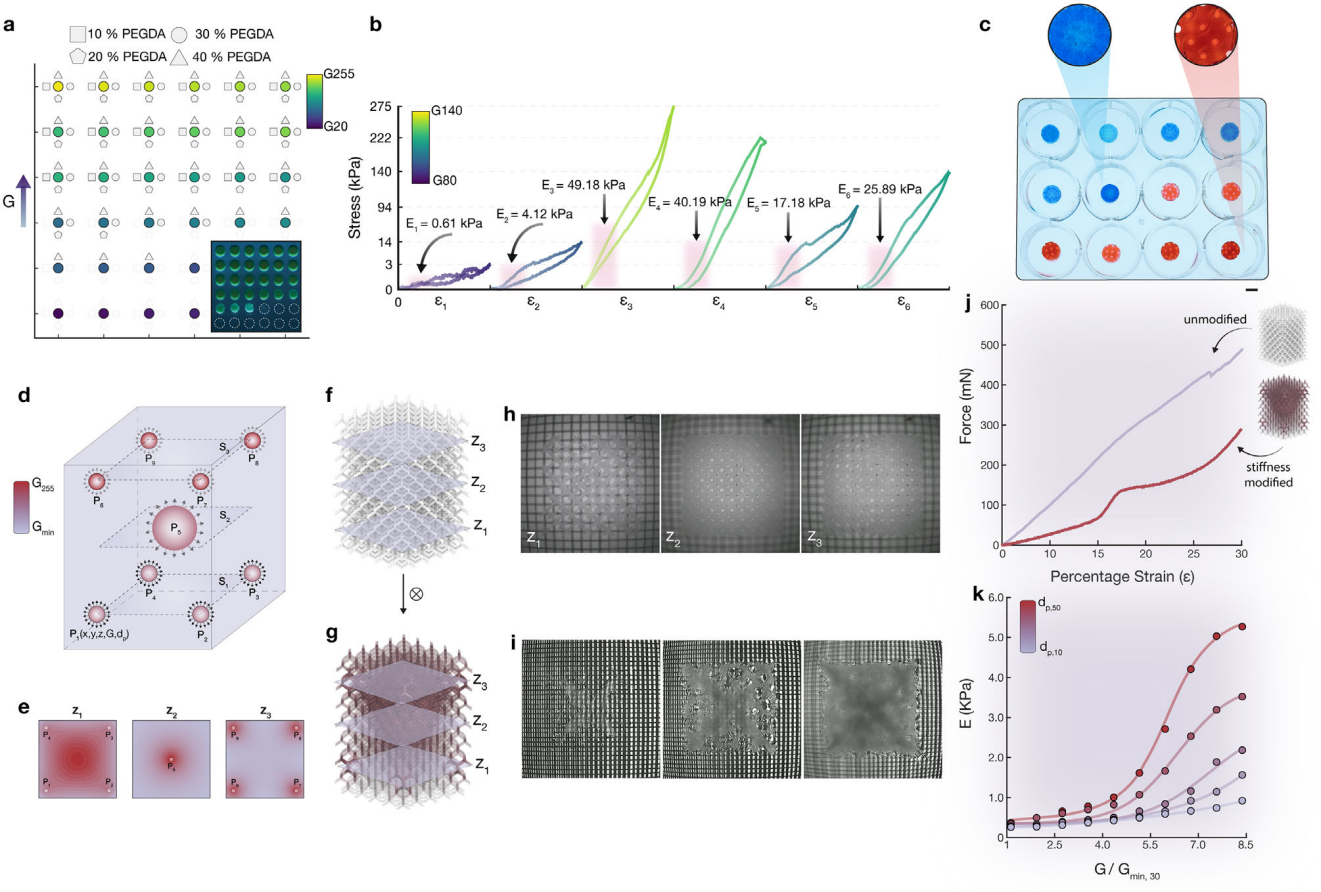
Stiffness modulation and three‐dimensional greyscale lithography. (a) Parameter space analysis of greyscale lithography for four distinct PEGDA material combinations, demonstrating the correlation between geometric feature fidelity and applied greyscale intensity. (b), Demonstration of iterative stiffness optimization for a 35% poly(ethylene glycol) diacrylate (PEGDA) gyroid lattice structure targeting a global elastic modulus of 25 kPa. Sequential iterations were fabricated in individual wells of a multi‐well plate. (c), Split‐P and Gyroid architectures fabricated within a single 12‐well plate, with red and blue dyes incorpdesign approach. orated for visualization. (d), Schematic representation of the three‐dimensional nodal framework for volumetric greyscale intensity distribution. (e), Representative cross‐sectional planes illustrating the spatial greyscale distribution. (f), Fluorite reference geometry with indicated nodal plane positions. (g), Volumetric greyscale intensity distribution generated through the convolution of the target geometry with the three‐dimensional nodal point cloud. (h), Sequential cross‐sectional images of the fluorite reference geometry at designated z‐positions. (i), Sequential cross‐sectional images of the greyscale‐modified fluorite geometry at corresponding planar positions. (j), Compressive force‐strain relationships for fluorite structures in both unmodified and greyscale‐modified configurations. k, Resultant elastic modulus of the fluorite structure as a function of decay strength (*D_p_*) and greyscale ratio (*G* / *G*_*min*,30_).

To address this, we present an automated approach for evaluating grayscale lithography using a characterisation‐in‐the‐loop framework that leverages the in situ fabrication and DIP's high volumetric throughput capabilities. For each material, a grayscale parameter map is first established to define the effective working range of grayscale values that yield resolvable features under a fixed optical power (Figure 3a). As such, this parameter map establishes the minimum and maximum optical dosage that can be applied to achieve a target structural modulus. This enables us to subsequently optimise greyscale parameters to achieve a desired structural modulus, without any prior knowledge of the structure's topology, material parameters or the material's opto‐mechanical response. Here we employ a simple learning approach based on zero‐order optimization (also referred to as Continuum‐armed bandits’ optimization) [78, 79], where the goal is to optimise an unknown objective function *f*(*x*), given oracle access to evaluations at adaptively chosen inputs *x*. This approach treats the grayscale parameter space as a continuous‐armed bandit problem where each grayscale value represents an “arm” with an unknown reward function (negative modulus error), employing an upper confidence bound (UCB) strategy through controlled exploration that balances exploitation of learned gradient information with exploration of unknown gradient information. Beginning with an initial grayscale guess, the algorithm uses measured modulus from each printed specimen to estimate the local gradient (∂*E*/∂*g*) via finite differences, updating this estimate through an exponential moving average with confidence weighting. For the next iteration, the algorithm employs a Newton‐Raphson control scheme that predicts the optimal grayscale adjustment *g*_*k* + 1_, falling back to proportional control when gradient confidence is low, while adding controlled Gaussian noise that decays exponentially with iteration count to implement the bandit exploration strategy. A full description of this approach is outlined in the methods section.

To demonstrate this optimization strategy, we apply the characterisation‐in‐the‐loop approach to iteratively determine the grayscale value required to fabricate a cylindrical gyroid triply periodic minimal surface (TPMS) structure with a target bulk modulus of 25 kPa, directly within a multi‐well plate. The initial grayscale input *g*_1_ is selected from the lower bound of the material's grayscale parameter map. The scaffold is printed and its effective modulus is measured, after which the model predicts the subsequent greyscale input *g*_*k* + 1_; this process is repeated until convergence. Convergence accuracy was quantified as the percentage modulus error at each iteration *k*. Across independent optimization runs (*n* = 5) for a given structure type, the algorithm typically converges within 4–6 iterations, reaching a target modulus within 3%–5% accuracy across the evaluated material formulations and structural designs. To highlight this process, a Gyroid comprising 35% PEGDA with a 4 mm unit cell size was sequentially printed within a multi‐well plate, achieving a final target modulus of 25.89 ±  0.91 KPa (mean ± s.d., *n* = 5; Figure 3b). An example of the resultant stiffness‐seeking approach for an additional 4 mm Gyroid comprised of 20% PEGDA (red) and a 4 mm unit cell Split‐P structure comprised of 8:10% PEGDA/Gelatin co‐polymer (blue) is shown in Figure 3c. Wherein the Split‐P construct had a target stiffness of 12 kPa (12.39 ± 0.67 KPa,  mean  ± s.d.,  *n*  =  5) and the Gyroid construct had a target stiffness of 25 KPa (25 ± 0.89 /KPa,  mean  ± s.d.,  *n*  =  5).

In addition to the global greyscale patterning demonstrated thus far, it is often advantageous to locally dictate the greyscale behaviour volumetrically in three‐dimensions. Whereby, localised stiffness variations can be used to produce structures that exhibit far greater mechanical complexity than can be achieved by global patterning alone. Therefore, to enhance the spatial resolution of greyscale patterning beyond uniform global exposure, we implemented a three‐dimensional point cloud methodology that permits the volumetric generation of greyscale masks for any arbitrary geometry. This approach establishes a bounding box volume encompassing the target geometry, within which a discrete set of nodes is distributed. Each node is characterised by its spatial coordinates (x,  y,  z), an assigned greyscale intensity value (G), and an exponential decay parameter (*d_p_*) that governs the spatial attenuation of greyscale influence *p*(*x*, *y*, *z*, *G*, *d_p_*) (Figure 3d). The resultant greyscale distribution at any position within the bounding box is computed through the cumulative contribution of all nodes, where the maximum grayscale value is G_max_ = 255. Cross‐sectional representations of this nodal framework, as illustrated in Figure 3e, demonstrate the spatial distribution arising from nine discrete nodes with heterogeneous greyscale values and decay parameters. Following the establishment of this three‐dimensional greyscale template, convolution with the voxelized target geometry yields a spatially modulated 3D greyscale distribution (Figure 3f,g).

Leveraging the real‐time imaging capabilities inherent to DIP, we directly visualized the fabricated structures in situ, enabling the visualization of greyscale crosslinking density variations through detection of local refractive index modulations in the polymerized network (Figure 3h,i). To demonstrate the influence of volumetric greyscale modulation on both bulk mechanical properties and the engineering of nonlinear mechanical response, we fabricated paired structures—unmodified and greyscale‐modified—composed of 8% (w/v) (PEGDA) and 10% (w/v) Gelatin employing a ruthenium/sodium persulfate (Ru/SPS) photo‐initiating system (Figure 3j). Unlike Type I photoinitiators (e.g., Irgacure 2959, LAP), which undergo direct unimolecular photocleavage under UV irradiation. Ru/SPS is a Type II photoinitiating system in which photoexcited Ru(II) undergoes electron transfer to SPS, generating sulfate radicals. Importantly, in this context, these sulfate radicals selectively oxidise tyrosine residues to form covalent dityrosine bonds [80, 81], enabling crosslinking of unmodified (pristine) gelatin without the need for chemical pre‐functionalisation such as methacrylation [82]. Utilising this material composition, an unmodified control structure exhibits a linear stress‐strain relationship throughout the loading regime. In contrast, we demonstrate that a greyscale‐modified structure shows a biphasic mechanical response characterised by an initial compliant deformation phase, followed by pronounced strain‐stiffening behaviour as progressive recruitment of the heterogeneous structural elements occurred under continued loading.

To systematically investigate and establish correlations between nodal parameters and the resultant structural modulus, we conducted a parametric analysis under fixed nodal positions. To constrain the parametric search scope and to investigate how nodal quadrants effect structural response, we employed a simple 9‐node arrangement, with 8 of the nodes placed at 25% in from each of the eight corners with a central nineth node, with this arrangement subsequently applied to a 2 mm UC fluorite lattice. The input parameters *G* and *d_p_* were systematically varied, with the minimum greyscale value maintained at *G_min_* = 30 to ensure structural reproduction, and the nodal decay constant ranging from *d_p_* =  30 − 50 (Figure 3k). As anticipated, under conditions of minimal greyscale variability (i.e., *G*  ≈  *G_min_*), the decay behaviour of individual nodes exhibited negligible influence on the final structural stiffness, with low values of *D_p_* yielding an approximately linear correlation with increasing *G*. However, with increasing exponential decay constant *d_p_*, a sigmoidal relationship emerged for increasing *G*, wherein the inflection point demonstrated tunability towards lower greyscale values through modulation of the effective influence radii of individual nodes on the global greyscale distribution. While the volumetric greyscale distribution described here is relatively simple, such an approach in combination with in situ characterisation provides the potential to design and validate arbitrary mechanical behaviour by modulation of both morphology and local stiffness gradients.

### 2.4 On the Fly Reconstruction

**FIGURE 4:**
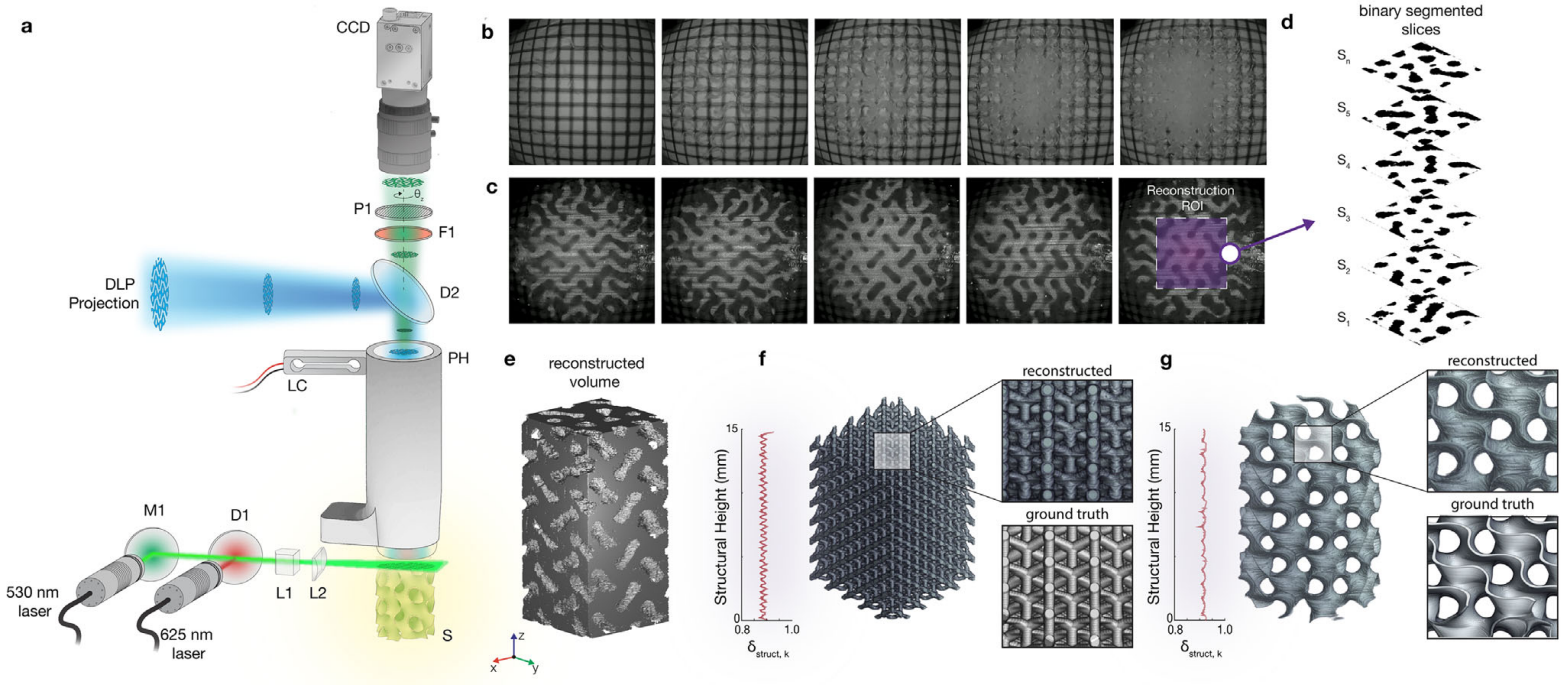
On‐the‐fly volumetric reconstruction. (a), Schematic depiction of the light‐sheet imaging setup used for on‐the‐fly volumetric reconstruction. Two laser sources (λ  =  530 *nm*,  λ  =  625 *nm*) are combined using a mirror (M1) and dichroic mirror D1 and further shaped using a powell lens (L1) and cylindrical lens (L2). A further colour filter (F1) and linear polarizer (P1) are used to segment the chosen wavelength and prevent unwanted back reflections from the DLP illumination. A final dichroic mirror (D2) is used to permit the illumination and coaxial imaging of the print domain simultaneously. (b), Time series printing of a Gyroid lattice (UC = 2 mm) under standard brightfield backlight illumination. (c), Time series printing of a Gyroid lattice (UC = 2 mm) under light‐sheet illumination. (d), Image stack of segmented regions corresponding to the ROI in c, with the structural regions and corresponding background indicated as white and black respectively. (e), Corresponding reconstructed volume for the ROI shown in (c). (f), Volumetric reconstruction of a Fluorite lattice (UC = 2 mm), with left inset showing the relative error between the reconstructed slice and the ground truth slice via SSIM. The right insets show a visual comparison between the reconstructed region and the ground truth (input mask). (g), Volumetric reconstruction of a Gyroid lattice (UC = 4 mm), with left inset showing the relative error between the reconstructed slice and the ground truth slice via SSIM. The right insets show a visual comparison between the reconstructed region and the ground truth (input mask).

While a purely brightfield imaging configuration can give an indication of structural quality, it inherently captures a superposition of all fabricated regions within a single image. This presents significant challenges for quantifying structural quality during fabrication, as isolating local structural features from the global cumulative field becomes problematic. To address this limitation, we implemented an orthogonal light‐sheet imaging pathway relative to the print direction, enabling segmentation of layer‐wise cross‐sections during fabrication (Figure 4a). The system comprises dual laser sources operating at 530 nm (green) and 625 nm (red), selected to match specific fluorophore excitation requirements. These sources were combined into a single beam path using a right‐angle mirror (M1) and a dichroic mirror (D1). Beam shaping is achieved through a Powell lens (L1), with subsequent focusing via a cylindrical lens (L2) positioned at the print field centre. A spectral filter (F1) is incorporated to transmit only fluorescent emission to the CCD sensor, while a linear polarizer (P1) suppresses back‐reflected illumination from the projection system.

To demonstrate the advantages of light‐sheet illumination for real‐time structural imaging, we fabricated a 2 mm gyroid lattice unit cell containing rhodamine B and compared imaging under brightfield illumination (Figure 4b) and light‐sheet excitation (λ  =  530 nm, Figure 4c). Unlike brightfield imaging, light‐sheet illumination enables accurate layer‐wise segmentation of the target structure, permitting the inspection and quantification of the internal architecture in real‐time. The technique generates cross‐sectional images with inherent contrast arising from refractive index differences between polymerized structures and surrounding prepolymer, as shown in Movie S2. While these cross‐sections can theoretically be extracted through binary segmentation, light‐sheet propagation through the entire structure introduces scattering and attenuation artifacts that can be challenging to segment via conventional image segmentation algorithms based on global thresholding, edge detection or region growth, which are sensitive to intensity variation, noise and local contrast variations. To mitigate these challenges, here we employ a machine learning segmentation approach, based on the open‐source Segment Anything Model (SAM 2) [83]. This model was further fine‐tuned on a small dataset of manually segmented video frames, produced by our light‐sheet system under various lighting and structure morphologies. This approach achieves accurate layer‐wise segmentation despite noise, distortion, and limited foreground‐background contrast (Figure 4d). Real‐time volumetric reconstruction is therefore achieved by integrating segmented binary cross‐sections along the print direction (Figure 4e).

To evaluate reconstruction performance across representative geometries, we fabricated and imaged two lattice architectures using the light‐sheet system: a fluorite lattice (unit cell, UC = 2 mm; Figure 4f) and a gyroid lattice (UC = 4 mm; Figure 4g). For each target cross‐section, reconstruction accuracy was quantified using the structural similarity index (SSIM) between the SAM2‐predicted binary segmentation and the ground‐truth binary projection, yielding a layer‐wise structural error δ_struct, *k*_. The overall reconstruction error for each structure, δ_struct_ was then defined as the mean of δ_struct, *k*_ across all frames (see Methods). Using this metric, the Fluorite and Gyroid lattices achieved reconstruction accuracies of 86.33 ±  4.47% and 91 ±  3.21%, respectively (mean  ± s.d.,  *n*  =  3).

## 3 Discussion and Future Perspectives

This work presents a high‐throughput integrated platform for mechanical and volumetric optical characterisation of hydrogel materials using DIP, permitting in situ characterisation within a fluid medium directly in standard labware (e.g., multi‐well plates), eliminating the need for sample transfer of soft/delicate biological materials. This reduces characterisation time and potential for structural damage. Using our characterisation platform, we develop a computationally efficient approach for optimizing bulk structural stiffness through bulk optical intensity modulation, requiring no prior knowledge of topology, material composition, or intensity‐stiffness relationships. Additionally, to explore the effect of local greyscale modulation, we introduce a simple volumetric greyscale methodology that generates arbitrary volumetric greyscale distributions based on three‐dimensional bounding box nodal weights, applicable to any topology. Parametric analysis of this approach reveals both linear and nonlinear mechanical behaviours achievable through designed greyscale modulation.

Further, to extend real‐time imaging capabilities, we incorporated a light‐sheet illumination system and a corresponding image processing framework based on SAM2, which predicts structure boundaries from a learned representation of the spatial context of light‐sheet‐illuminated regions, rather than relying on explicit intensity thresholds or edge heuristics. This results in substantially improved robustness to shadowing, illumination gradients, scattering, and measurement noise. Nonetheless, performance is still expected to degrade under severe attenuation, scattering, or strong domain shift relative to the training distribution [84]. While the initial SAM2 training dataset was constructed with examples of structures directly relevant to this study, future work may be required to expand the dataset to more diverse, generalized geometries to improve reconstruction performance and robustness across a wider range of structural classes. Future implementations could be used to dynamically update projections and corresponding greyscale distributions in real‐time to permit closed‐loop control of structural reproduction.

As approaches are developed that can transition bio fabrication from the lab‐bench to industrial scale utility, the capacity to develop process analytical technologies (PAT) [85, 86] for bioprinting is crucial. Such frameworks enable fabrication to be controlled against measured quality attributes, supporting the standardization not only across independent trials and batches, but importantly across independent laboratories. This is critical for normalizing variability arising from materials, operators, system configurations, and process conditions before drawing biological or functional conclusions from printed constructs. Here, we implement such an approach based on the evaluation of DIP‐printed materials, with the real‐time light‐sheet reconstruction component demonstrated for semi‐transparent objects at approximately the centimetre scale. While stiffness‐seeking itself is unaffected by a resin's optical properties, the use of bioinks with high concentrations of optically scattering components (e.g., cells, particles), opaque materials, or larger cross‐sections may hinder volumetric reconstruction, requiring the incorporation of index‐matching additives or alternative reconstruction strategies.

In conclusion, we demonstrate a DIP‐based structural evaluation platform that permits both the mechanical evaluation and real‐time volumetric reconstruction of printed structures in situ. Whilst the work that we have demonstrated utilises DIP as the fabrication approach, future implementations could explore how in situ mechanical characterisation could be translated to alternative printing modalities, including volumetric printing, Xolography [58], CLIP [65, 66] and light‐based fabrication generally.

## 4 Methods

### 4.1 Dynamic Interface Printing Setup

The experimental DIP configuration employed a commercial projection system (LRS‐WQ, Visitech) equipped with a 2× projection lens, yielding a lateral resolution of 15.1 µm at the focal plane. The system delivered variable irradiance levels ranging from 0 to 270 mW cm^−^^2^ at a peak wavelength of 405 nm, facilitating polymerization across diverse material formulations and photoinitiator concentrations. Real‐time process monitoring was enabled through integration of a coaxial illumination system coupled with a monochrome CCD affixed to a variable zoom long working distance microscope lens, permitting direct visualization and spatial registration during fabrication. Sample positioning was achieved using a custom core‐XY translation stage for lateral motion and a dedicated linear actuator for vertical displacement. Print head pressure regulation was implemented through a motorized syringe drive coupled with an inline pressure sensor (MMR902A34A, MITSUMI), providing quantitative feedback for maintaining optimal interface conditions throughout the fabrication process. System integration was achieved using a commercial motion controller (Octopus V1.1, BIGTREETECH), with coordinated control of motion planning, projection timing, and pressure regulation executed through a custom MATLAB interface.

### 4.2 Mechanical Characterisation Setup

Integration of mechanical characterisation capabilities within the fabrication platform was achieved through incorporation of cantilever‐based force transducers positioned adjacent to the print head aperture. Two load cells with nominal capacities of 100 g (SEN‐14727, SparkFun Electronics) and 500 g (SEN‐14728, SparkFun Electronics) were implemented based on measurement requirements. Signal acquisition from the force transducers was performed using 24‐bit ADC (HX711, Adafruit) connected to an Arduino Uno microcontroller, which permitted force, displacement and duration data to be acquired over serial within our MATLAB control interface. This configuration enabled continuous force monitoring, load cell calibration, hydrostatic pressure calibration, and contact point detection prior to mechanical characterisation. Print head components were fabricated using stereolithography (Form 3+, Formlabs) with Tough 1500 photopolymer resin.

### 4.3 Load Cell Calibration

The force data utilised in this work was acquired via a 24‐bit ADC, and as such, the load‐cell output is initially uncalibrated and must be converted from raw ADC counts to force through calibration. Accordingly, prior to each experimental run, we performed a calibration using a set of traceable test masses (TMCS, Nuweigh) spanning the operating range of each sensor. For the 100 g load cell, 10 masses from 1 to 100 g were used; for the 500 g load cell, 10 masses from 1 to 500 g were used. An unloaded measurement was also recorded to define the zero‐load offset.

As the load‐cell response is approximately linear with applied load (±0.05% full scale), we fit a linear regression relating ADC counts to force:

$$F=g\left(\alpha \cdot D+\beta \right)$$

Where ***F*** denotes the force, ***g*** denotes the gravitational constant, ***α*** is the scale factor (g/count), ***D*** is the raw ADC counts and ***β*** is the offset (zero‐load bias). To validate the calibration, an additional pre‐calibrated mass not included in the calibration dataset was measured and independently verified using a laboratory microbalance (ABS 320–4N, KERN).

### 4.4 Load Correction Under Hydrostatic Conditions

Because structures are mechanically evaluated in situ within a liquid bath immediately after fabrication, hydrostatic contributions during uniaxial compression can confound the measured force response, due to the relative displacement of the fluid volume caused by print head insertion. This effect is particularly pronounced for low‐modulus materials, where the hydrostatic force may be comparable to, or in some cases exceed, the structural load. Accordingly, prior to fabrication, the system performs a hydrostatic (density) calibration by translating the print head through a known vertical interval [***z***_***bottom***_, ***z***_***top***_] (typically 25% of the container height) while recording paired samples ${\left\{\left(z_{i},\;F_{i}\right)\right\}}_{i=1}^{N}$. Over this interval, the hydrostatic contribution varies linearly with depth; therefore, linear regression of the sampled data yields

$$\hat{F}\;\left(z\right)=\hat{\beta_{0}}+\hat{\beta_{1}}z$$

where $\hat{F}\left(z\right)$ denotes the predicted hydrostatic force at depth ***z***, $\hat{\beta_{0}}$ is the force offset at ***z***  =  0, and $\hat{\beta_{1}}$ is the hydrostatic gradient given by

$\beta_{1}\approx \rho g\;\frac{dV}{dz}=\rho gA_{p}$ where ***ρ*** is the fluid density, ***g*** is the gravitational constant, ***V*** is the displaced volume, and ***A***_***p***_ is the effective wetted cross‐sectional area of the print head. During uniaxial compression, the structural force is obtained by subtracting the hydrostatic component from the measured force:

$\;F_{struct,\;j}=F_{meas,j}\;-\left(\hat{\beta_{0}}+\hat{\beta_{1}}z_{j}\right)$

### 4.5 Greyscale Parameter Bounding

As the stiffness‐seeking algorithm modulates greyscale intensity to converge on a target structural modulus, it is important to define appropriate boundary conditions prior to its application. Establishing these bounds restricts the search space to regions of the greyscale parameter domain that yield resolvable structures, thereby excluding conditions that would result in either insufficient polymerization or overcuring.

For a fixed exposure intensity and duration, a matrix of cylindrical features was projected onto a coverslip containing the selected hydrogel formulation over a range of greyscale values. The resulting features were subsequently imaged and quantified relative to the target geometry, yielding a binary parameter map that delineates resolvable and nonresolvable regions as a function of greyscale intensity (Figure 3a). The lower and upper bounds derived from this map were then used to constrain the parameter search space employed by the stiffness‐seeking algorithm.

### 4.6 Determination of Effective Structural Modulus

The effective structural modulus was computed using the apparent footprint area of the fabricated construct. For a cylindrical scaffold, the footprint area, engineering strain, and engineering stress are defined as, $A_{0}=\pi {\left(\frac{D}{2}\right)}^{2}$, $\varepsilon =\frac{\delta_{h}}{H_{0}}$, and $\sigma =\frac{F_{struct}}{A_{0}}$. Where ***D*** denotes the diameter of the structure for cylindrical TPMS geometries, ***δ***_***h***_ denotes the change in height of the structure, ***H***_0_ denotes the original height, and ***F***_***struct***_ is the recorded structural force after hydrostatic compensation. The effective modulus was then calculated as the slope of the stress–strain curve over a defined strain interval:

$$\;E_{\mathrm{eff}}={\left.\frac{d\sigma }{d\varepsilon }\;\right|}_{\varepsilon \in L}$$

Where L denotes the selected strain window. Here, L was chosen as 5–10% strain to avoid nonlinearity associated with the initial structural contact whilst remaining below the onset of geometry‐driven nonlinear effects such as buckling and snap‐through.

### 4.7 Stiffness Seeking Approach

The following outlines the algorithmic approach for stiffness seeking, wherein the goal is to determine an optimal greyscale intensity ***g*** *, such that the recorded bulk structural modulus matches that of the target modulus. As the relationship between the greyscale intensity and the resulting material properties is typically nonlinear [87, 88], as the polymerization rate ***R***_***p***_ is given by,

$$\;R_{p}=k_{p}\;\left[M\right]\sqrt{\frac{R_{i}}{2k_{t}}},\;\mathrm{where}\;R_{i}=2f_{0}\phi \alpha \left[PI\right]I_{0}e^{-\alpha \left[PI\right]L/2}$$

Where, ***k***_***p***_ denotes the propagation rate, [***M***] is the monomer concentration, ***k***_***t***_ is the termination rate, ***f***_0_ is the initiator efficiency, ϕ is the quantum yield, ***α*** is the initiator absorption, [***PI***] is the initiator concentration, ***I***_0_ is the optical intensity and ***R***_***i***_ is the local rate of initiation at the position ***x***  =  ***L***/2 inside the reaction medium.

As such, this nonlinear behaviour in addition to measurement noise, directly modelling this inverse relationship is challenging. As such, we employed a simple approach to sequentially iterate the greyscale intensity, followed by subsequent mechanical characterisation, to quickly determine the optimal greyscale intensity parameters to achieve a desired stiffness. The approach is as follows:

For a given target modulus ***E***_***target***_, we want to find the greyscale value $g^{*}\in \left[G_{\min},\;G_{\max}\right]$ such that

$$\;g^{*}=\arg\min_{g\;\in \left[G_{\min},\;G_{\max}\right]}\left|f\left(g\right)-E_{target}\right|\;$$

where $f:\left[G_{\min},\;G_{\max}\right]\to R^{+}$ is the unknown material response function which maps the corresponding greyscale intensity to the bulk structural modulus and ***G***_***min***_ and ***G***_***max***_, correspond to the input boundary conditions from the greyscale parametric sweep. In the absence of a parametric sweep, these values can simply be replaced by [1,  255], at the expense of convergence speed. The approach is first initialized either by taking the mid‐point of the greyscale bounds, or the upper or lower bound, or a known starting condition based on previous test data. This greyscale value therefore, establishes ***g***_0_, where the primary control scheme can be broken into three distinct sections.

### 4.8 Gradient Estimation

We Establish a Running Estimate of the Local Gradient $\nabla f\approx \;\frac{\partial E}{\partial g}$ Using Finite Differences From Recent Measurements

$$\nabla \;f_{k}=\frac{1}{n}\sum_{i=1}^{n}\frac{E_{i}-E_{i-1}}{g_{i}-g_{i-1}}$$

where ***n*** denotes the number of recent measurements in the iteration loop, with ***E***_***i***_ and ***g***_***i***_ denoting the corresponding recorded modulus and projected greyscale intensity, respectively. The gradient estimate prediction for the next iteration was correspondingly updated using an exponential moving average

$$\nabla \;f_{k+1}=\left(1-\alpha \right)\;\nabla f_{k}+\alpha \nabla f_{new}$$

where α∈[0,1] denotes the algorithm learning rate and ∇ ***f***_***new***_ is the newly calculated gradient estimate from the most recent measurement, prior to being incorporated into the running estimate ∇ ***f***_***k***_. We further established a confidence metric $c_{k}\;\in \left[0,\;1\right]$, which is used to track the reliability of the gradient estimate, with

$$\;c_{k+1}=\min\left(1,\;c_{k}+\Delta c\right)$$

where **Δ** ***c*** is the confidence increment per successful gradient update. This control scheme is used to decide whether to trust the current gradient estimate or to fall back on a simpler control strategy. For example, in early iterations where we have few data points, the gradient estimate is therefore unreliable or for regions of nonlinearity wherein old gradient estimates are invalid.

### 4.9 Adaptive Control

We further employed two simple control strategies contingent on the gradient estimate confidence. When the gradient confidence is high (**c**_**k**_ > **c**_**min**_, and ∇**f**_**k**_ > ε), we employed a Newton‐like stepping scheme using the estimated gradient to determine the next predicted greyscale value, with

$\;g_{k+1}=g_{k}+\frac{E_{target}-E_{k}}{\nabla f_{k}}$

When the Gradient Estimate Is Low, We Employ a Simple Proportional Control Scheme With Gain ***λ***, With

$\;g_{k+1}=g_{k}+\lambda \;\frac{E_{target}-E_{k}}{E_{target}}$

### 4.10 Exploration‐Exploitation Balance

To Prevent Local Minima and Improve Gradient Estimation, We Also in some Cases Utilized an Additional Exploration Scheme

$$\;g_{k+1}=g_{predicted}+\sigma_{k}\cdot N\left(0,\;1\right)$$

where N(0,1) denotes a standard normal distribution of ***σ***_***k***_ = ***σ***_0_  · exp (− ***k***/***τ***), where ***k*** is the current iteration with exponential decay constant ***τ***, where N(0,1) represents a random number drawn from this distribution. As such, the likelihood of model exploration therefore decays for increasing iteration count.

### 4.11 Convergence Criteria

The Algorithm Terminates either When ***k*** > ***k***_***max***_ Corresponding to the Iteration Count Exceeding a Predetermined Upper Iteration Bound ***k***_***max***_ or When

$\frac{|E_{k}-E_{target}|}{E_{target}}\;\leq \varepsilon_{tol}$

Denoting that the measured modulus ***E***_***k***_ falls within the target modulus ***E***_***target***_ to within a predefined tolerance range ε_***tol***_ (normally <5%).

### 4.12 Generation of Volumetric Greyscale Structures

To produce spatially variable material properties within a single construct based on modulating the local exposure intensity, we established a 3D greyscale field ***G*** (***x***), where ***x***  =  (***x***, ***y***, ***z***) denote the spatial coordinates of the field. The goal here is to define a smooth and continuous resultant greyscale field from a set of finite control points by first modelling the multi‐point influence across all nodes, followed by multiplicative field composition and finally obtaining the resultant greyscale field via volume convolution with the target input geometry. As such, we denote a control node ***p***_***i***_ =  (**x**_**i**_, ***y***_***i***_, ***z***_***i***_), with a greyscale value $G_{i}\in \left[0,\;255\right]$, and corresponding decay constant ***d***_***p***, ***i***_ > 0. As such, the influence of point ***i*** at location ***x*** follows an exponential decay

$$w_{i}\;\left(x\right)=\exp\left(-\frac{∥x-p_{i}{∥}_{2}}{d_{p,i}}\right)\;$$

where larger values of ***d***_***p***, ***i***_ correspond to reduced nodal decay, permitting the extension of a given nodal value across a greater extent of the control volume. While here we use an exponential decay rate, which guarantees ***C*** ^∞^, other decay functions can also be used (e.g., linear, log, or polynomial). Therefore, the complete greyscale field emerges from the multiplicative combination of all control points' influences, where

$G\;\left(x\right)=G_{0}\;\prod_{i=1}^{n}{\left(\frac{G_{i}}{G_{0}}\right)}^{w_{i}\left(x\right)}$

Here, ***G***_0_ represents the background greyscale value, typically set to ***G***_***min***_ to permit a minimum threshold of the field to match that of the material's response. The ratio ***G***_***i***_/***G***_0_ acts as a scaling factor, with values greater than unity increasing the local exposure of a given field position, with the exponentiation by ***w***_***i***_(***x***) representing the interpolation of this scaling effect based on the proximity to the nodal position.

Depending on the size of the required field and the number of control points, it is therefore often computationally prohibitive to compute the entire field at full resolution. In this instance, we employed a two‐stage approach, where the field is computed at a smaller subset of the target resolution, followed by a tri‐cubic interpolation to map the subset field to the full‐resolution field to match that of the target geometry. Finally, we convolved the two fields together to determine the corrected target geometry based on the greyscale field template

$$V\;\left(x\right)=G\left(x\right)\cdot I\left(x\right)$$

where ***V*** (***x***) denotes the final corrected greyscale volume, whose binary topological inputs are given by ***I*** (***x***).

### 4.13 Light Sheet Imaging Setup

The dual‐wavelength light sheet imaging system was constructed using green (530 nm, 250 mW) and red (660 nm, 300 mW) laser modules. The beams were combined via a right‐angle mirror (Edmund Optics #45‐595) and green transmissive dichroic filter (Edmund Optics #66‐247). Beam expansion was achieved using a 30° Powell lens (Edmund Optics #43‐473), followed by focusing through a 150 mm focal length cylindrical lens (Edmund Optics #46‐200) positioned to create the light sheet at the centre of the print container. The resulting light sheet thickness, measured at the focal plane using a CCD camera, was approximately 40 µm. To minimize backscattered illumination from the projector and enable selective fluorophore detection, a glass linear polarizer (Edmund Optics #43‐787) and appropriate coloured glass filters were integrated into the camera's optical path upstream of the microscope objective.

### 4.14 Volumetric Reconstruction

Conventional segmentation methods proved inadequate for processing cross‐sectional images acquired by the light sheet imaging system due to scattering artifacts, optical aberrations, and acquisition noise. To address these limitations, we implemented a machine learning segmentation approach using the Segment Anything Model 2 (SAM 2) [83] architecture. To optimize performance for our specific imaging conditions, we fine‐tuned the pre‐trained SAM 2 model using a curated dataset of manually annotated frames from the coaxial imaging system. This training dataset encompassed diverse structure types and illumination conditions representative of our experimental range. Each training sample consisted of an image frame paired with its corresponding manually generated binary segmentation mask. The fine‐tuned SAM segmentation model could therefore be run in real‐time, with segmented regions integrated in the printing direction to reconstruct the three‐dimensional volume.

### 4.15 Volumetric Quantification

To quantify the discrepancy between the ground‐truth geometry and the reconstructed volume obtained via light‐sheet segmentation, we used the structural similarity index (SSIM) computed between the ground‐truth binary cross‐section and the corresponding binary segmentation predicted by SAM2. For each layer ***k***, the layer‐wise structural error was defined as

$$\;\delta_{\mathrm{struct},\;k}=\mathrm{SSI}M_{k}\;\in \left[0,1\right]$$

To avoid intersecting the meniscus surface and introducing scattering artefacts during acquisition, the light sheet was positioned ***D***_***LS***_ =  500 µ**m** below the projection plane. This axial offset introduces an index lag between the projected slice and the recorded slice that depends on the layer height ***D***_***LH***_:

$$\;S_{r,\;k}=S_{k-\;\frac{D_{LS}}{D_{LH}}}$$

Where, ***S***_***r***, ***k***_ denotes the recorded slice and ***S***_***k***_ denotes the projected slice. Accordingly, each captured light‐sheet frame was compared to the corresponding ground‐truth cross‐section at $k-\frac{D_{\mathrm{LS}}}{D_{\mathrm{LH}}}$ after image registration, Therefore, each captured light‐sheet frame is compared against the corresponding cross‐section at $k-\;\frac{D_{\mathrm{LS}}}{D_{\mathrm{LH}}}$, by first applying image registration, and then computing **SSIM**_***k***_ to obtain ***δ***_**struct**, ***k***_. This procedure was repeated for all frames in the sequence, and the total reconstruction error was defined as the mean layer‐wise error:

$\;\delta_{\mathrm{struct}}=\frac{1}{K}\sum_{k=1}^{K}\delta_{\mathrm{struct},\;k}$

All structures were sliced with a layer height of 50 µm over a 15 mm build height (***K***  =  300 frames per structure), with ***n***  =  3 independent replicates per structure.

### 4.16 Model Preparation

All TPMS and lattice structures were generated in nTop, converted to a triangulated mesh and exported as an STL file. For each geometry, the STL file was loaded into a custom slicing interface developed on the open‐source Slic3r framework [89], which facilitated geometric manipulation and positioning within the standard labware (e.g., cuvettes, multi‐well plates) used for this work. Structures were subsequently sliced into a PNG image‐stack, which was then imported into our MATLAB GUI interface to perform convex slice correction, prior to printing.

### 4.17 Material Synthesis

#### 4.17.1 PEGDA‐Based Materials

Various concentrations of PEGDA were used in this study, ranging from 10% to 35% w/v. For each formulation, we followed the same protocol. The required weight fraction of poly(ethylene glycol) diacrylate (PEGDA, Mn 700) (455008, Sigma) was dissolved in the corresponding volume fraction of 40°C deionized water and thoroughly mixed for 10 min. Subsequently, 0.035 w/w% of tartrazine (T0388, Sigma) and 0.25% w/w of LAP (900889, Sigma) were added to the mixture and stirred until complete dissolution. The materials were then stored in light‐safe Falcon tubes until required.

#### 4.17.2 PEGDA/Gelatin Co‐Polymer

The PEGDA/gelatin copolymer was formulated with 8% (w/v) poly(ethylene glycol) diacrylate (PEGDA, Mn 700) and 10% (w/v) cold‐water fish gelatin (G7041, Sigma‐Aldrich). Gelatin was dissolved in deionized water preheated to 40°C under continuous agitation on a rocker plate until complete solubilization was achieved. Subsequently, PEGDA was incorporated into the gelatin solution at the specified concentration. Photocrosslinking was facilitated through a ruthenium/sodium persulfate (Ru/SPS) dual‐component photoinitiating system (#5248, Advanced Biomatrix), which enables simultaneous covalent crosslinking of tyrosine residues on gelatin chains and acrylate groups on PEGDA backbones. The photoinitiator concentrations were maintained at 0.8 mM ruthenium and 8 mM sodium persulfate. This concentration, consistent with the 1:10 ratio reported in previous studies employing Ru/SPS [16, 90, 91], is higher than that typically used in volumetric printing applications, where elevated concentrations (e.g., above ∼0.5 mM/5 mM [Ru/SPS]) can compromise optical penetration [92, 93]. However, unlike volumetric printing—where light must traverse the entire volume (≳1 cm) unobstructed to ensure uniform dosage—our approach requires light penetration of only ∼10–50 µm during fabrication. This relaxed optical constraint allows us to leverage the higher photoinitiator concentration to enhance crosslinking response and improve shape fidelity.

As the ruthenium complex is inherently red and therefore by itself partially blocks blue/uv light, a low concentration of tartrazine (T0388, Sigma‐Aldrich) was incorporated as a photoabsorber at 0.025% (w/w) to further prevent undesired curing through the material depth. The complete formulation was vortexed until uniform dispersion was achieved. Air bubbles introduced during sequential mixing were removed by centrifugation at 2000 rpm for 5 min. Final solutions were stored in light‐safe Falcon tubes and utilized within 24 h of preparation to prevent premature crosslinking via redox‐initiated sulfate radical formation from ruthenium‐catalyzed persulfate decomposition.

#### 4.17.3 Dying Agents

To enhance visualization of the hydrogel constructs, blue (Pillar Box Blue Food Colour, Queen) and red (Pillar Box Red Food Colour, Queen) food colouring were used. Printed structures were immersed in a concentrated aqueous dye solution (30 µL mL^−1^) for 30–60 min prior to imaging.

## Conflicts of Interest

The co‐authors have an interest in a start‐up (www.cymphonybio.com) that is commercializing dynamic interface printing technologies.

## Supporting information




**Supporting File 1**: advs74708‐sup‐0001‐Movie S1.mp4

**Supporting File 2**: advs74708‐sup‐0002‐Movie S2.mp4

## Data Availability

The data that support the findings of this study are available from the corresponding author upon reasonable request.
